# Midkine Prevents Calcification of Aortic Valve Interstitial Cells *via* Intercellular Crosstalk

**DOI:** 10.3389/fcell.2021.794058

**Published:** 2021-12-15

**Authors:** Qian Zhou, Hong Cao, Xiaoyi Hang, Huamin Liang, Miaomiao Zhu, Yixian Fan, Jiawei Shi, Nianguo Dong, Ximiao He

**Affiliations:** ^1^ Department of Physiology, School of Basic Medicine, Tongji Medical College, Huazhong University of Science and Technology, Wuhan, China; ^2^ Center for Genomics and Proteomics Research, School of Basic Medicine, Tongji Medical College, Huazhong University of Science and Technology, Wuhan, China; ^3^ Hubei Key Laboratory of Drug Target Research and Pharmacodynamic Evaluation, Huazhong University of Science and Technology, Wuhan, China; ^4^ Department of Cardiovascular Surgery, Union Hospital, Tongji Medical College, Huazhong University of Science and Technology, Wuhan, China

**Keywords:** CAVD, scRNA-seq, cell communication, VICs’ calcification, midkine (MDK)

## Abstract

Calcified aortic valve disease (CAVD), the most common valvular heart disease, lacks pharmaceutical treatment options because its pathogenesis remains unclear. This disease with a complex macroenvironment characterizes notable cellular heterogeneity. Therefore, a comprehensive understanding of cellular diversity and cell-to-cell communication are essential for elucidating the mechanisms driving CAVD progression and developing therapeutic targets. In this study, we used single-cell RNA sequencing (scRNA-seq) analysis to describe the comprehensive transcriptomic landscape and cell-to-cell interactions. The transitional valvular endothelial cells (tVECs), an intermediate state during the endothelial-to-mesenchymal transition (EndMT), could be a target to interfere with EndMT progression. Moreover, matrix valvular interstitial cells (mVICs) with high expression of midkine (MDK) interact with activated valvular interstitial cells (aVICs) and compliment-activated valvular interstitial cells (cVICs) through the MK pathway. Then, MDK inhibited calcification of VICs that calcification was validated by Alizarin Red S staining, real-time quantitative polymerase chain reaction (RT-qPCR), and Western blotting assays *in vitro*. Therefore, we speculated that mVICs secreted MDK to prevent VICs’ calcification. Together, these findings delineate the aortic valve cells’ heterogeneity, underlining the importance of intercellular cross talk and MDK, which may offer a potential therapeutic strategy as a novel inhibitor of CAVD.

## Introduction

Calcified aortic valve disease (CAVD), the most prevalent form of aortic valve stenosis, affects approximately 3% of the population aged over 60 years, and so far, it has lacked pharmacological treatment (Osnabrugge et al., 2013). Previously considered a degenerative disease, CAVD is now thought to be an active cellular process driven by intricate cell-to-cell interactions with complex mechanisms. The progression of the disease includes three phases: 1) initial endothelial dysfunction and injury, 2) low-density lipoprotein cholesterol deposition, and 3) immune cell infiltration, oxidative stress, and pro-inflammatory cytokine stimulations (Akat, Borggrefe, and Kaden 2009; Li, Xu, and Gotlieb 2011; Towler 2013). Such changes give rise to innate and adaptive immune cell infiltration, valvular endothelial cells’ (VECs’) transformation, and valvular interstitial cells’ (VICs’) activation, leading to a complicated aortic valve microenvironment. Moreover, cell-to-cell interactions are closely related to the maintenance of normal aortic valve physiological functions and the development of CAVD. For instance, macrophages and VICs communicate with VICs through IL6 and CDH11 molecules, respectively, to accelerate the osteogenic differentiation of VICs (Hutcheson et al., 2013; Grim et al., 2020). Comparing VICs and VECs’ co-culture in the osteogenic medium with only VICs in the osteogenic medium, it was found that expressions of osteogenic markers (RUNX2 and αSMA) in the co-culture medium were reduced, indicating that VECs inhibited the VICs’ calcification (Akat, Borggrefe, and Kaden 2009). Although cell–cell interactions were examined from bulk RNA-seq and experimental data, there was a lack of comprehensive and systematic research on cell heterogeneity and cell-to-cell communication in CAVD.

Recently, single-cell RNA sequencing (scRNA-seq) technologies have allowed the identification of calcified-associated cell types and trends of cell fate, and provided unprecedented details of the valve heterogeneity and interactions among cell subpopulations (Xu et al., 2020).

In this study, we characterized the cell types with functional states of biological relevance. There were two special and novel cell subsets. First, the VECs’ subset was an intermediate state in the process of VECs to VICs’ transformation, that is, in the process of endothelial-to-mesenchymal transition (EndMT), so they are named transitional valvular endothelial cells (tVECs). Second, the T-cell subset was derived from a normal aortic valve and involved in extracellular matrix (ECM) organization, which has never been reported. Furthermore, we systematically analyzed cell-to-cell interactions mediated by ligand–receptor interactions across all cell subsets in the aortic valve microenvironment. Intriguingly, matrix valvular interstitial cells (mVICs) highly expressed midkine (MDK) and mainly interacted with activated valvular interstitial cells (aVICs) and compliment-activated valvular interstitial cells (cVICs) through MDK-NCL ligand–receptor. Subsequently, we validated that MDK inhibited calcification of VICs that calcification by Alizarin Red S staining, real-time quantitative polymerase chain reaction (RT-qPCR), and Western blotting assay *in vitro*. In summary, we determined the functional status of each cell type, VICs’ heterogeneity, and intercellular cross talk among all cell subsets. We identified that MDK prevented VICs’ calcification as well, which provided a potential therapeutic target for CAVD treatment.

## Materials and Methods

### Gene Expression Data

The scRNA-seq data of two normal and four calcific samples were sequenced by our group (Xu et al., 2020). The sample collection and harvesting of single cells were described in detail in the study by Xu et al. (2020). Briefly, after the operation, these valves were separated and washed with cold l × PBS, and then mechanically dissociated using eye scissors. Dissociated samples were digested in DMEM with collagenase type I, 2 mg/ml (Sigma-Aldrich, Saint Louis, MO) to prepare a single-cell suspension because the extracellular matrix of the aortic valve is mainly composed of type I collagen that completely degrades extracellular matrix. Healthy aortic valve tissue specimens were harvested from patients undergoing repair of aortic dissection requiring aortic valve replacement. The standards for healthy aortic valves included the following: 1) heart color Doppler echocardiography showed no obvious thickening of valve leaflets and no nodules; 2) histopathological examination showed no calcium nodules. The data also can be downloaded from the GEO database with accession number PRJNA562645. Microarray data of human aortic valves were downloaded from the GEO database with accession number GSE51472 and microarray data of human VICs were downloaded from the GEO database with accession number GSE88803.

scRNA-seq matrix: https://www.jianguoyun.com/p/DRQVbtkQp8_2CRiVrZME

scRNA-seq annotation: https://www.jianguoyun.com/p/DX5htpQQp8_2CRjZrJME.

Bulk RNA-seq data: https://www.jianguoyun.com/p/DbnawFYQp8_2CRjyq5ME.

Experimental data: https://www.jianguoyun.com/p/DRV3dbEQp8_2CRiDrJME.

### scRNA-Seq Analysis and Identification of the Major Cell Types

Raw gene expression matrices of scRNA-seq data were integrated and regenerated to a Seurat object by the Seurat R package (version 3.2.0) (Stuart et al., 2019). Cells with fewer than 2000 UMIs, over 6,000 or below 500 expressed genes, over 20% UMIs derived from mitochondrial genome, and log10 UMIs of per gene lower than 0.8 were removed. We then used the NormalizeData function to normalize the library size of each cell with default parameters and the FindVariableFeatures function to select the top three thousand genes that are the most variably expressed genes of each sample. The FindIntegration Anchors and IntegrateData functions were used to integrate data with default parameters, which made a normalized Seurat object. Principal components analysis was performed and the first 30 PCs were used to further generate t-SNE dimensionality reductions of the RunTSNE function. Graph-based clustering was run using FindNeighbors and FindClusters functions with a resolution of 0.3. Cell clusters were annotated using canonical marker genes.

### Gene Set Variation Analysis

To estimate the purity of diverse cell subsets in the aortic valve from bulk RNA-seq and scRNA-seq data, we calculated stromal and immune scores using single-sample gene set enrichment analysis (ssGSEA) (Barbie et al., 2009). Stromal and immune gene sets are from curated datasets in ESTIMATE software (Yoshihara et al., 2013). To assign pathway activity of individual cell subset, we used GSVA (Hanzelmann, Castelo, and Guinney 2013) with standard parameters. Immune and stromal score data: https://www.jianguoyun.com/p/DSpzs1gQp8_2CRiGrJME.

### Differential Expression Genes Analysis

Microarray data were normalized and log2-transformed using the limma R package (version 3.44.1) (Ritchie et al., 2015). We then used this package to filter the differential expression genes between disease and normal samples. The cutoff thresholds: adjusted *p* values <0.05 and |log2fold change (FC)| > 1. For scRNA-seq data, in order to compare the transcriptional characteristics of normal and calcified groups in the major cell types, we used the FindMarkers function (Seurat v3) (Stuart et al., 2019) to screen the differential expression genes from each group in the three major cell types. The cutoff thresholds: in the calcified group or normal group, the gene is expressed in more than 25% of cells, adjusted *p* values <0.05, log2fold change (FC) > 0.5.

### Gene Ontology Analysis

Gene Ontology (GO) analysis was performed using Metascape (https://metascape.org/) (Zhou et al., 2019). REVIGO (Supek et al., 2011) was used to remove redundant GO terms. Ultimately, top 5 pathways were remained to visualize.

### Subclustering of the Major Cell Types

We performed integrated analyses of three main cell types so as to subdivide the subsets of each main cell type. The number of PCs was determined by dataset specificity. Based on the graph-based clustering approach of the FindClusters function, the resolution of VECs, immune cells, and VICs was 0.4, 0.2, and 0.2, respectively. For visualization purposes, these informative PCs were converted into t-SNE plots as above. Then, the FindAllMarkers function was used to define the expression of corresponding marker genes.

### Trajectory Analysis

Pseudotime ordering of VECs was performed using monocle 2 (version 2.16.0) (Trapnell et al., 2014). Differential gene expression analysis used the differentialGeneTest function. Dimensional reduction and cell ordering were performed using the DDRTree method and orderCells function.

### Transcription Factors Analysis

Motifs of transcriptional factors were found by the SCENIC package (version 1.1.3) (Aibar et al., 2017). Transcriptional factors of hg19 as a reference were downloaded using RcisTarget. Gene regulatory networks were inferred using GENIE3 and according to the gene expression matrix of each cell subset.

### Cell-To-Cell Communication Analysis

To investigate potential interactions among cell subsets in the aortic valve microenvironment, cell-to-cell communication analysis was performed using CellChatDB (Jin et al., 2021) and CellChat R package (version 0.0.2).

### Cell Culture and Processing

Isolation and culture of VICs follow the previous method (Zhou et al., 2020). The experiments were divided into four groups, namely, the control group with the DMEM containing 2% fetal bovine serum (FBS), VICs cultured in OM (Cyagen Biosciences, HUXMA-90021), the MDK group supplemented with 100 ng/ml MDK(PEPROTECH,450-16), and both OM and MDK.

### Calcification Analysis

Alizarin Red S staining was used to evaluate the degree of osteogenic differentiation of VICs. Cells were plated on a 12-well cell culture plate at a density of 30,000/cm^2^. Before treatment, starving with serum-free medium for 24 h, the four groups were cultured for about 21 days. After the treatment, the cell culture plate was washed twice with phosphate-buffered saline (PBS) solution, fixed with 4% paraformaldehyde (PFA) for 10 min, and then stained with 2% Alizarin Red S for 30 min at room temperature. After dyeing, it was rinsed with deionized water three times.

### Western Blot Analysis

After the cells were cultured for five days, the protein was collected by RIPA containing protease inhibitors. After the protein concentration was measured by the kit, a quarter volume of the loading buffer (Servicebio, G2013) was added, denaturing at 95°C for 10 min. The protein samples were resolved by SDS-PAGE (4–20% gels) and then transferred to PVDF membranes using a protein transfer instrument (eBlotL1, GENSCRIPT). After 15 min of QuickBlock™ Western blocking solution (Beyotime, P0252) at room temperature, the membrane was incubated overnight with the primary antibody at 4°C for RUNX2(CST, 8486s), ALP (Zenbio,220,678), and GAPDH (Proteintech,60004-1-I). qRT-PCR assay using Cell Total RNA isolation kit (FOREGENE, RE-O3113) was utilized to extract mRNA. HiScript III RT SuperMix (Vazyme, R323-01) was used to perform reverse transcription of mRNA. Then, the reverse transcription product was used as a template to perform qRT-PCR on a StepOnePlus thermal cycler (Applied Biosystems, Foster City, CA) using ChamQ Universal SYBR qPCR Master Mix (Vazyme, Q711-02) to analyze the difference in gene expression. The primers used were designed at the NCBI and synthesized by Tsingke Biological technology.

Primer.

**Table:** 

Homo ALP-F	GAC​AAA​CTG​GGG​CCT​GAG​ATA
HOMO ALP-R	CTG​ACT​TCC​CTG​CTT​TCT​TGG
HOMO GAPDH-F	GAG​AAG​GCT​GGG​GCT​CAT​TT
HOMO GAPDH-R	AGT​GAT​GGC​ATG​GAC​TGT​GG
HOMO RUNX2-F	GCG​CAT​TCC​TCA​TCC​CAG​TA
HOMO RUNX2-R	GGC​TCA​GGT​AGG​AGG​GGT​AA

### Statistical Analysis

To determine statistical significance in the analysis of immune score and stromal score for each sample, we used a two-sided Student’s *t*-test with Bonferroni correction. Statistical significance between multiple samples was determined using a one-way analysis of variance (ANOVA). All statistical analyses were used R (version 4.0.2).

## Results

### Major Cell Types Are Identified in Aortic Valve Microenvironment

**FIGURE 1:**
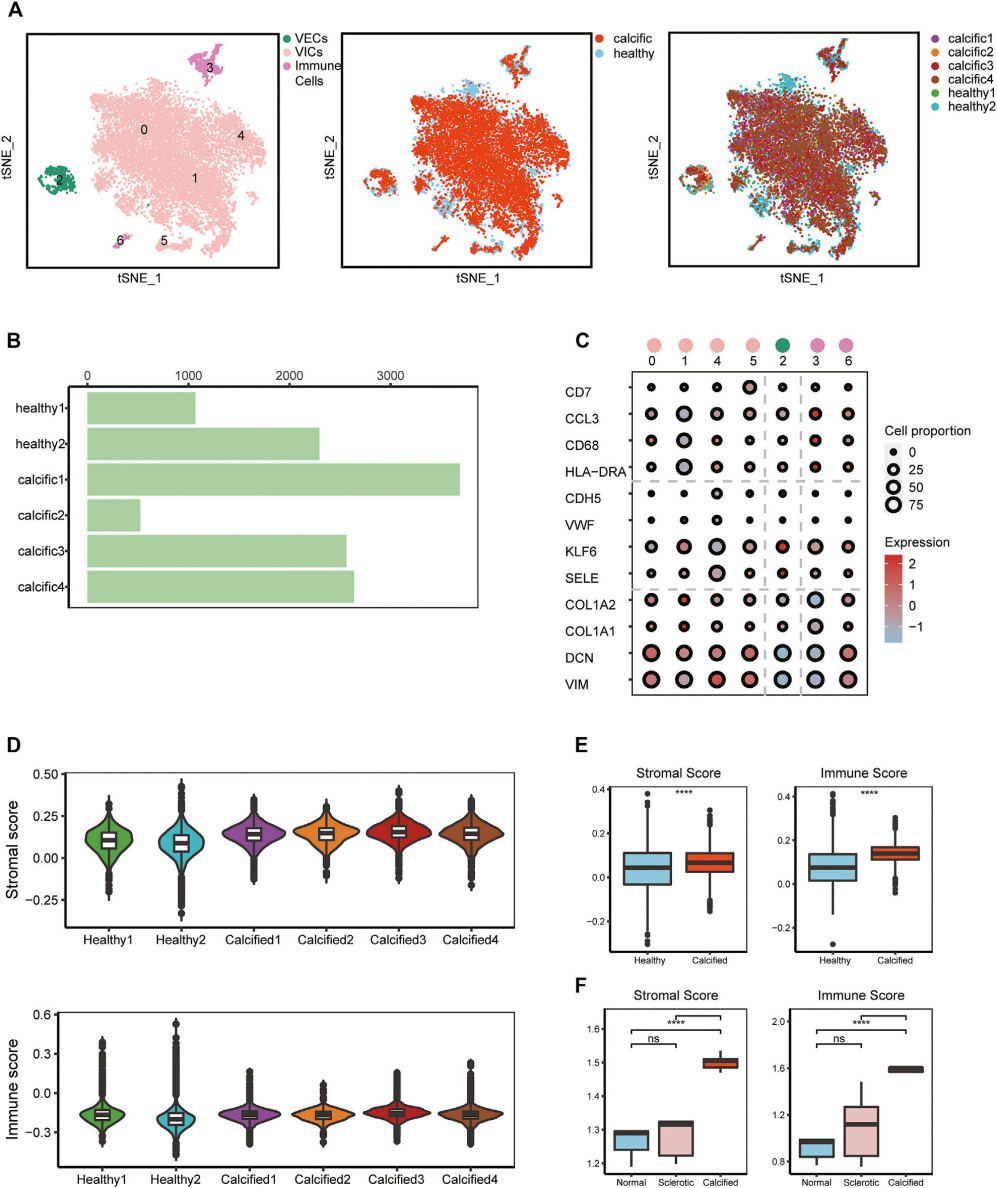
scRNA-seq analysis identifies major valve cell types**. (A)** Specific cell types were defined (left); t-SNE plot colored by disease groups (middle); every cluster contains cells from normal and calcified patients (right). **(B)** Cell numbers of each sample. **(C)** Canonical markers of VECs, VICs, and immune cells; the dot size represents the number of cell proportions. **(D–F)** Stromal score and immune score in all samples from scRNA-seq and bulk RNA-seq data (n = 5, per group). Box plots describe the median and interquartile range (IQR) of each score. The whiskers depict the 1.5 x IQR. *p < 0.05, **p < 0.01, ***p < 0.001, ****p < 0.001.

To investigate cellular heterogeneity and cell-to-cell interactions in aortic valves microenvironment at single-cell resolution, we used scRNA-seq data previously from our research group, including two normal and four calcific samples (Xu et al., 2020) (Supplementary Figure S1). After stringent filtering (Supplementary Figure S2), 16,275 unique genes were obtained from 12,776 cells. Of these, 3,366 cells (26%) originated from normal samples and 9,410 cells (74%) from calcified samples (Figure 1B). Based on the known cell markers, there were three main populations in the aortic valve: immune cells (691 cells, 5.4%, marked with HLA-DRA, CD68, CCL3, and CD7); VECs (599 cells, 4.7%, marked with SELE, KLF6, VWF, and CDH5); VICs (11486 cells, 89.9%, marked with VIM, DCN, COL1A1, and COL1A2) (Figures 1A,C). There was no sample preference in different cell populations (Figure 1A), suggesting that batch effects were removed by the integrated analysis of scRNA-seq data.

To verify cell clustering, we reconfirmed features of major cell types based on scRNA-seq and bulk RNA-seq data. From the scRNA-seq data, for each main cell population, differential genes analysis was performed between calcific-derived and normal-derived cells (Supplementary Figure S3A-C). GO analysis, selecting upregulated genes of each calcific group, showed that VECs were involved in metabolic-related processes (Supplementary Figure S3D), immune cells were associated with immune-related processes (Supplementary Figure S3E), and VICs were connected with ECM remodeling processes (Supplementary Figure S3F). From bulk RNA-seq data, comparing calcific and normal tissues, there were 472 downregulated genes and 250 upregulated genes (Supplementary Figure S3G), of which upregulated genes were also involved in immune-related and ECM remodeling pathways (Supplementary Figures S3H,I). Considering ECM remodeling and immune activities were closely connected with valve calcification, we used stromal and immune scores to comprehensively estimate samples profiles. These two scores not only significantly increased in calcified samples from bulk RNA-seq data but also slightly raised in calcific samples from scRNA-seq data (Figures 1D,E). Consequently, we were convinced that all cell types were accurately identified and that three key cell populations existed in the aortic valve microenvironment, including VECs, VICs, and immune cells.

### The Environment Is Prominent in Shaping T-Cell Traits

**FIGURE 2:**
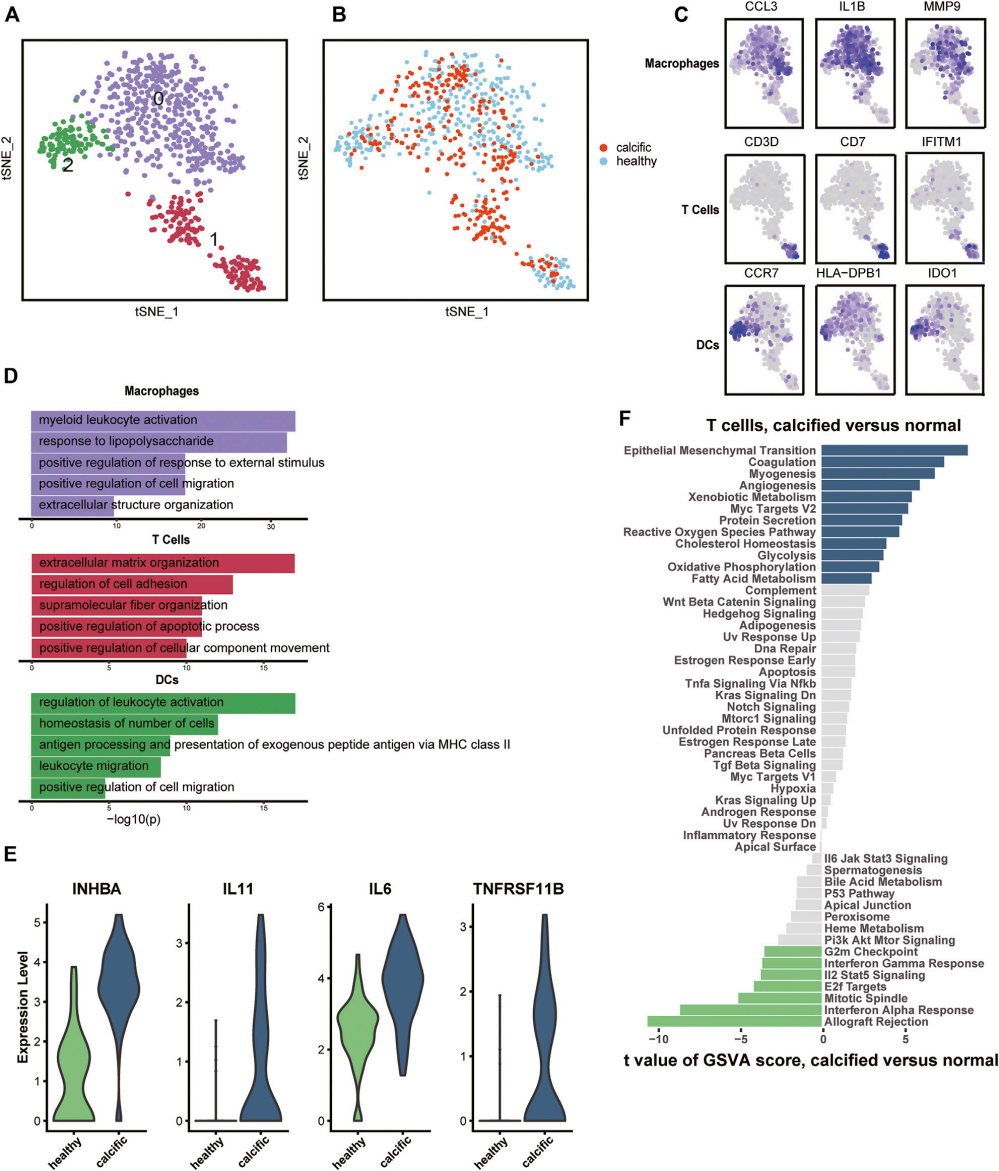
Immune cells’ profiles in the normal and calcified aortic valve. **(A)** Identification of macrophages, T cells, and DCs. **(B)** Colors display group of immune cells. **(C)** Marker genes: macrophages (CCL3, IL1B, and MMP9), T cells (CD3D, CD7, and IFITM1), and DCs (CCR7, HLA-DPB1, and IDO1). **(D)** Representative GO terms in each cluster. **(E)** Differential expression genes between normal-derived and calcific-derived T cells. **(F)** Gene enrichment of normal-derived and calcific-derived T cells in hallmark pathways.

Regarding inflammation and lipid infiltration, diverse immune cells infiltrate into the aortic valve, so we re-clustered immune cells from normal and calcific valves. The most immune cells were macrophages (IL1B, CCL3, and MMP9), followed by T cells (CD3D, CD7, and IFITM1) and were dendritic cells (DCs) (CCR7, HLA-DBP1, and IDO1) (Figures 2A,C, Supplementary Figure S4A). Macrophages and DCs activated leukocytes, which were the main features of early CAVD (Figure 2D). However, T cells had the ability to organize extracellular matrix, which aroused our curiosity (Figure 2D). To explore whether transcription factors caused biological differences, we used Single-Cell Regulatory Network Inference and Clustering (SCENIC) (Aibar et al., 2017) to evaluate the transcription factors activities of each subset. The activity of transcription factors varied greatly among cell types (Supplementary Figure S4B). The heatmap presented increased expressions of TFDP1 and RB1 in macrophages and DCs, while that of CEBPG, MECP2, and SAP30 decreased in the same clusters (Supplementary Figure S4B). In contrast, the expressions of these transcription factors showed an opposite trend in T cells (Supplementary Figure S4B).

Surprisingly, an interesting discovery in the t-SNE graph (Figure 2B) was that there was an evident dichotomy between normal-derived and calcific-derived T cells. This meant that T cells also resided in the normal aortic valve, which was not reported before. According to expression profiles, the genes relevant to calcification induction, such as INHBA, IL11, IL6, and TNFRSF11B, were specifically expressed in calcific-derived T cells (Figure 2E). Furthermore, EndMT pathways connected with CAVD were enriched in calcific-derived T cells (Figure 2F). These results suggested that T cells derived from distinctive environments promote aortic valve homeostasis or CAVD in some way.

### scRNA-Seq Reveals a Novel Cluster of VECs Correlated With EndMT in CAVD

**FIGURE 3:**
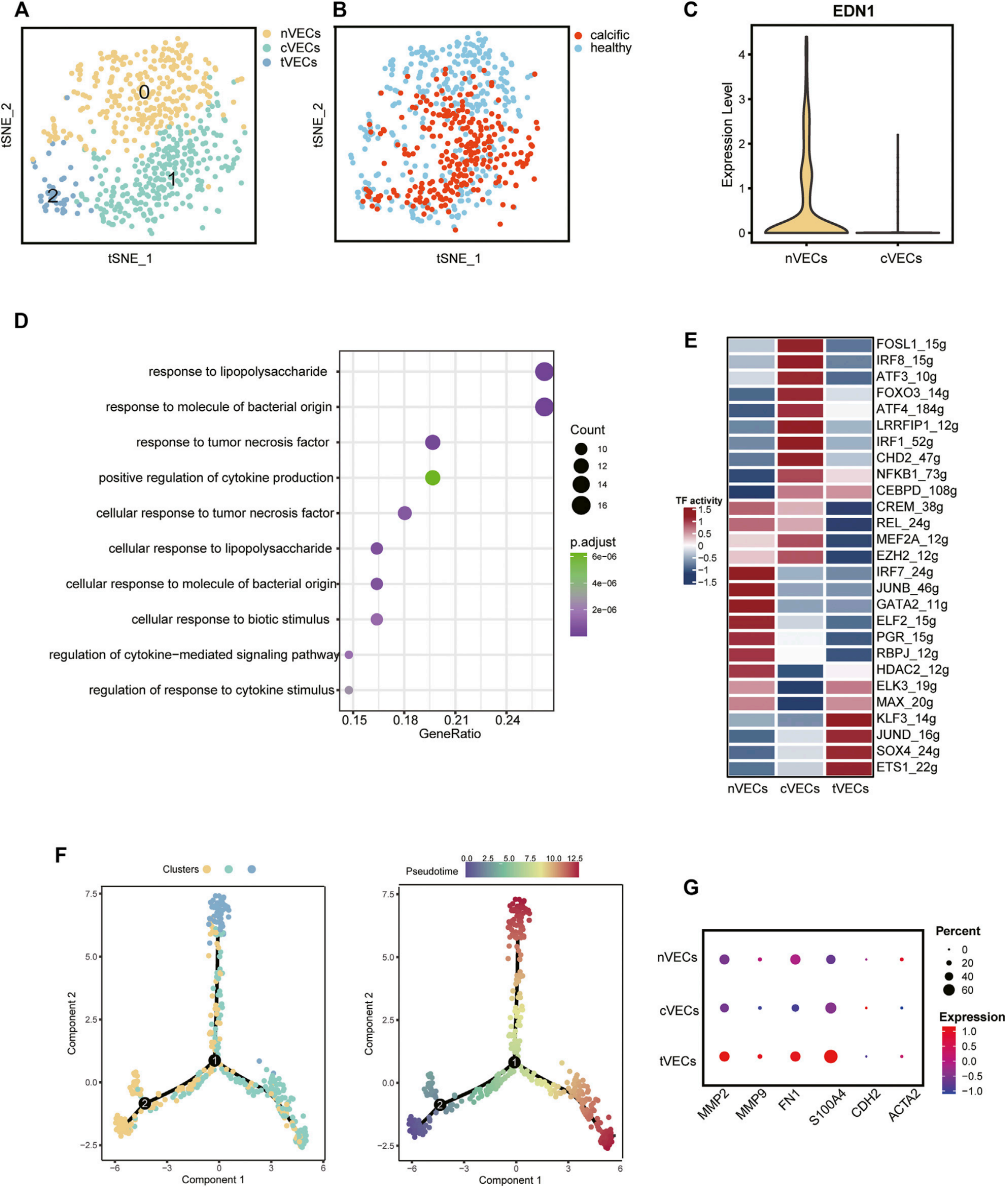
VECs’ profiles in normal and calcified aortic valve. **(A)** Main cell types of VECs. **(B)** Colors display the group of VECs. **(C)** The expression of EDN1 between nVECs and cVECs. **(D)** GO pathways of upregulated genes in cVECs compared with nVECs. **(E)** The heatmap shows the activity of transcription factors in each cell population. **(F)** Pseudotime analysis of VECs’ subsets. **(G)** Mesenchymal cell markers in VECs’ subsets, color representing expression, and dot size representing cell numbers.

VECs, from normal and calcific samples, were re-clustered. It was evident from the t-SNE graph that there were three subsets of VECs (Figure 3A). The largest number of cells was normal-derived VECs (nVECs) with high expression of END1, followed by calcified-derived VECs (cVECs), then a few cluster2 cells (Figures 3B,C). Comparing nVECs with cVECs, the noticeable enrichment of upregulated genes in nVECs was in response to tumor necrosis factor and cytokine stimulation (Figure 3D). On the other hand, transcription factors activity presented various profiles between these two populations. The expressions of IRF1, CEBPD, and NFκB1 increased in cVECs and those of JUNB and GATA2 decreased (Figure 3E). However, these transcription factors showed completely opposite expressions in nVECs.

EndMT driven by TGFβ, inflammation, and shear stress plays an essential role in the damage of valve endothelium in CAVD (Kovacic et al., 2019). Therefore, it was essential to explore the differentiation trajectory of VECs in EndMT by single-cell pseudotime analysis using monocle (Trapnell et al., 2014). The pseudotime analysis illustrated that nVECs were distributed at the beginning end of the pseudotemporal trajectory, whereas cVECs and cluster2 cells were located at the other two ends (Figure 3F). Interestingly, the expression of mesenchymal marker genes (MMP9, FN1, and S100A4) in cluster2 rose dramatically compared with other clusters (Figure 3G). Considering the features of cluster2 cells, we inferred that the cluster cells were in a transitional state from VECs to VICs, named tVECs. In conclusion, a novel cluster of VECs was found to exist in EndMT, which was regarded as a potential target for intervention in the process of conversion of VECs to VICs.

### VICs Exhibit a High Degree of Heterogeneity

**FIGURE 4:**
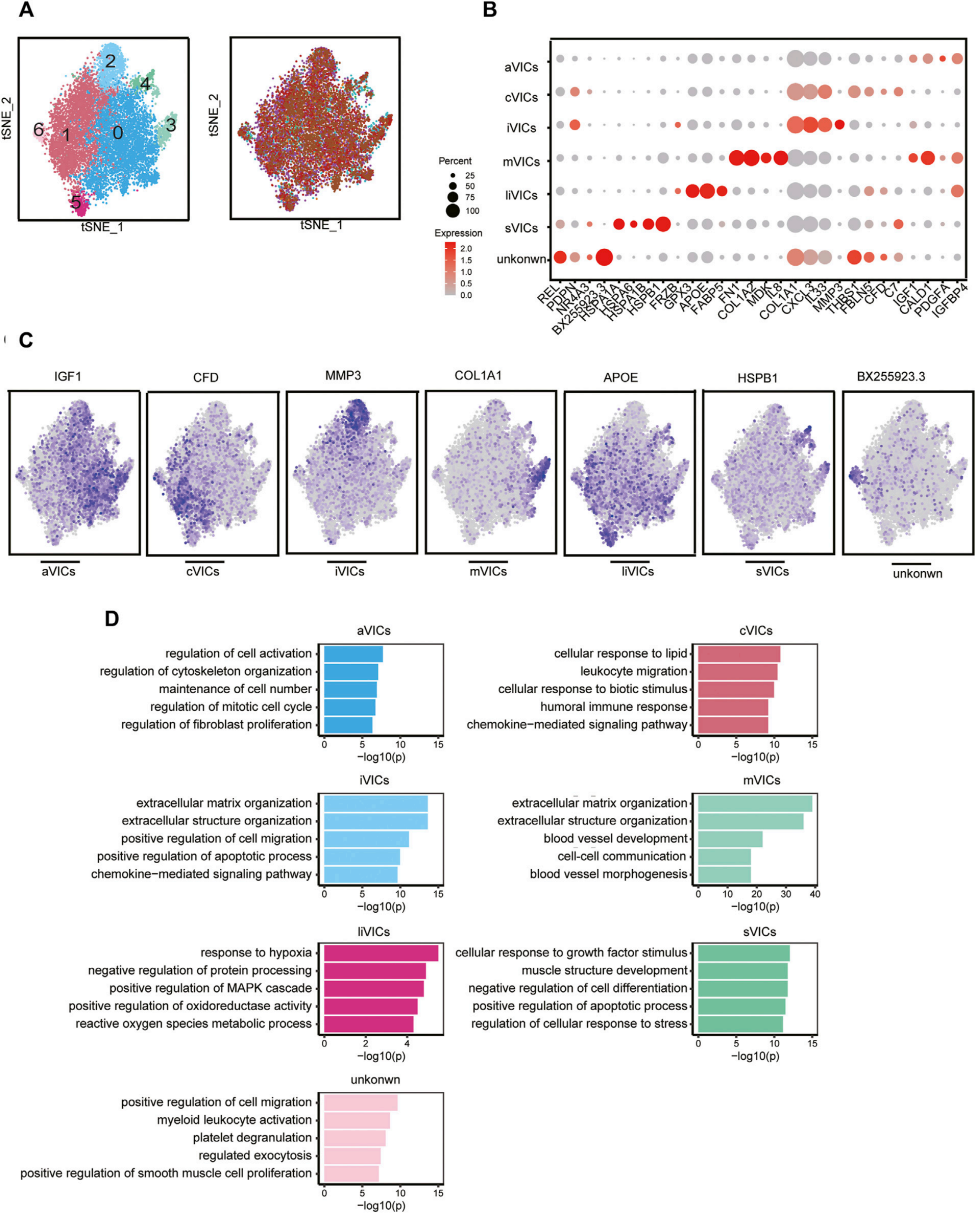
VICs heterogeneity. **(A)** VICs’ subsets colored by sample origin. **(B)** Markers expression of each cell subset. **(C)** Differential expression genes: aVICs (IGF1), cVICs (CFD), iVICs (MMP3), mVICs (COL1A1), liVICs (APOE), and sVICs (HSPB1). **(D)** GO terms of each subset.

CAVD is now considered to be an active disease process, mainly controlled by resident VICs (Rutkovskiy et al., 2017). Disease-induced stimuli transform VICs from quiescent fibroblast-like into active myofibroblast-like cells, thereby forming an intricate environment. Therefore, it is urgent to elucidate the heterogeneity of VICs in CAVD. We identified seven main subsets, of which cluster0 and cluster1 were the most abundant subsets, accounting for 78% (Figures 4A,C). Cluster0 VICs still expressed several myofibroblast-related genes (for example, IGF1, IGFBP4, CALD1, and PDGFA), which was in line with GO analysis showing cell activation and fibroblast proliferation (Figures 4B–D). These cells were termed aVICs. Cluster1 VICs presented gene signatures related to the cellular response to lipid and leukocyte migration (for example, C7 and CFD), which was also confirmed by GO analysis, so these cells were named cVICs (Figures 4B–D). Interestingly, both cluster2 and cluster3 VICs specialized in an extracellular matrix organization (Figure 4D). Cluster2 VICs were also involved in inflammatory responses except for remodeling ECM, with a signature expression of IL33, CXCL3, and MMP3, while cluster3 VICs only expressed stromal-related genes (for example, COL1A1, COL1A2, and FN1) (Figures 4B–D). Hence, the two clusters were termed inflammation-associated valvular interstitial cells (iVICs) and mVICs, respectively. Cluster4 VICs specifically expressed lipid metabolism-related genes (for example, FABP5, APOE, GPX3, and FRZB) and were named lipid-associated valvular interstitial cells (liVICs) (Figures 4B–D), suggesting that this cluster of VICs responded to lipid infiltration. The GO terms of this cluster were enriched in response to hypoxia and reactive oxygen species metabolism, which also confirmed our inference (Figure 4D). Cluster5 VICs, with characteristics of heat shock protein-related genes (for example, HSPB1, HSPA1B, HSPA6, and HSPA1A), were rich in the cellular response to growth factor stimulation and were named stress valvular interstitial cells (sVICs) (Figures 4B–D). However, cluster6 VICs were uncertain and we only observed that these cells may be related to inflammation (Figures 4B–D).

### Complex Intercellular Interactions in Aortic Valve Microenvironment

Cell-to-cell communication is an important regulator for maintaining the homeostasis of the aortic valves environment, including communication between the same cells and between different cells (Wang, Leinwand, and Anseth 2014). Therefore, we systematically explored potential communication among VICs, VECs, and immune cells.

**FIGURE 5:**
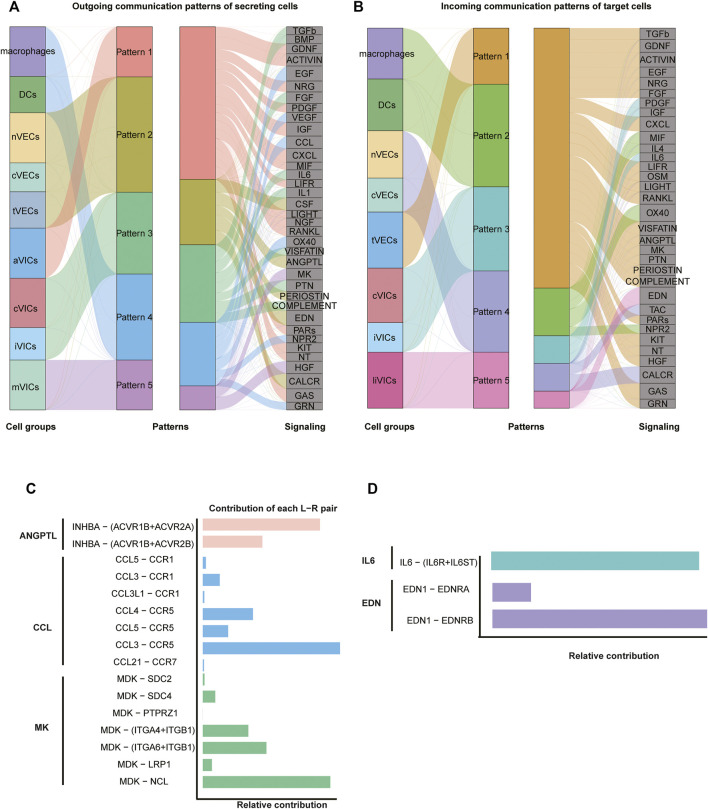
Intercellular communication. **(A)** Inferred outgoing communication, and the thickness of flow indicates the contribution of the cell population. **(B)** Inferred incoming communication. **(C,D)** Relative contribution of each pair of ligand–receptor.

Intercellular interactions were examined from two aspects of the outgoing signals (all cell subsets as senders) (Figure 5A; Supplementary Figure S5A) and the incoming signals (all cell subsets as receivers) (Figure 5B; Supplementary Figure S5B). Firstly, as for outgoing signals, the results showed that immune cells (including macrophages and DCs) sent signals through pattern4, which is a collection of many pathways mostly composed of CCL and EGF pathways (Figure 5A; Supplementary Figure S5B). VECs sent signals via pattern2, primarily including EDN, CALCR, and CSF approaches (Figure 5A; Supplementary Figure S5A). VICs sent out signals through three modes: pattern1, 3, and 5. That is, pattern1 was primarily composed of IGF, CXCL and GAS pathways; pattern3 consisted predominantly of FGF and PTN pathways; and pattern5 was primarily made up of MK and HGF pathways (Figure 5A; Supplementary Figure S5A). Secondly, from the perspective of afferent signals, immune cells received signals through pattern2 represented by the OX40 pathway (Figure 5B; Supplementary Figure S5B). VECs received signals through pattern4 represented by TAC and CALAR channels (Figure 5B; Supplementary Figure S5B). VICs received signals were pattern3 and pattern5, including CXCL, ACTIVIN and PDGF, and EDN channels, respectively (Figure 5B; Supplementary Figure S5B).

Since VICs play an important role in CAVD (Rutkovskiy et al., 2017), our investigation of cell communication focused on VICs: macrophages got in touch with VICs through CCL3 ligands and CCR5 receptors that belonged to the CCL pathway (Figure 5C; Supplementary Figures S6C,D). As for VECs, tVECs communicated with VICs through INHBA ligands and ACVR1B and ACVR2A receptors that were in the ANGPTL pathway (Figure 5C; Supplementary Figures S6A,B); nVECs interacted with VICs through EDN1 ligands and EDNRB receptors that were members of the EDN signaling pathway (Figure 5D; Supplementary Figures S7C,D). Correspondingly, EDN1 is also highly expressed in nVECs (Figure 3C). For communication among all VICs’ subsets, two interesting pathways were observed. aVICs and cVICs, as the main subsets secreting IL6 cytokines, affected other VICs’ subsets through paracrine (Supplementary Figures S7A,B). It was previously reported that macrophages secreted IL6 to affect VICs (Grim et al., 2020); however, here, we observed that activated VICs’ subsets could also secrete IL6 to induce other VICs’ subsets. In addition, cVICs released the inflammatory cytokine IL1 that had an impact on all VICs’ subsets (Supplementary Figures S6E,F).

### MDK From mVICs Inhibits VICs’ Calcification

**FIGURE 6:**
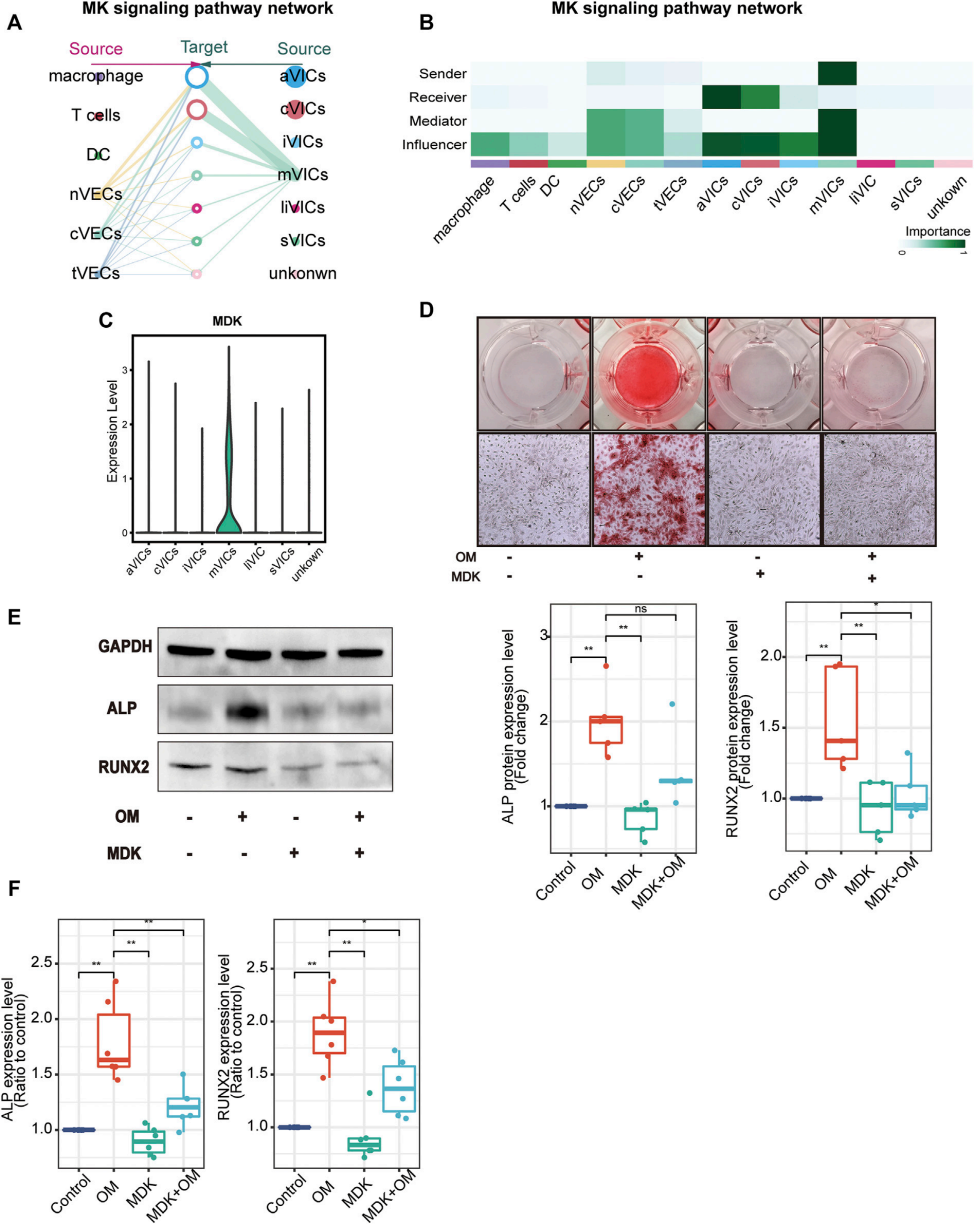
MDK inhibits VICs’ calcification**. (A)** The hierarchical plot shows the intercellular communication network of the MK pathway. The left portion describes the paracrine activity and the right portion describes autocrine activity; the solid and open circles represent the source and the target, respectively; the size of the circle represents the proportion of cell numbers in each cell subset; the width of the edge represents the communication probability; the edge color is the same as the source. **(B)** The relative importance of each subset with different roles. **(C)** The expression of MDK in VICs’ subsets. **(D)** Alizarin Red S staining of VICs with different treatments: control (normal culture medium), OM (osteogenic medium), MDK (normal culture medium-plus MDK treatment), and OM + MDK (osteogenic medium-plus MDK treatment). **(E)** The protein expression level of RUNX2 and ALP with different treatments (n = 5 per group). **(F)** The RNA expression level of osteogenesis-specific genes (RUNX2 and ALP) with different treatments (n = 6 per group). *p < 0.05, **p < 0.01.

After analyzing cell-to-cell interactions among all cell populations, we discovered a special signaling pathway MDK. This signal network indicated that MDK was only derived from mVICs and acted on all other VICs’ subsets in a paracrine manner, mainly aVICs and cVICs (Figures 6A,B). Moreover, MDK is significantly expressed in mVICs (Figure 6C). These results displayed that MDK had a key effect on VICs’ calcification. In order to better understand the influences of MDK in VICs’ calcification, relevant experiments were used for verification. VICs, isolated from aortic valve tissues of non-CAVD patients, were cultured in normal medium, osteogenic induced medium (OM), MDK-add medium, and MDK-add OM, respectively. Strikingly, there was no calcified nodule in all media except for OM, suggesting that MDK could reverse the formation of OM-induced calcified nodules (Figure 6D). In addition, regardless of the protein or transcription level, osteogenic differentiation markers RUNX2 and ALP were reduced in VICs after MDK treatment (Figures 6E,F), which was consistent with the above phenomena. Therefore, it was clearly indicated that MDK inhibited the osteogenic differentiation of VICs.

## Discussion

**FIGURE 7:**
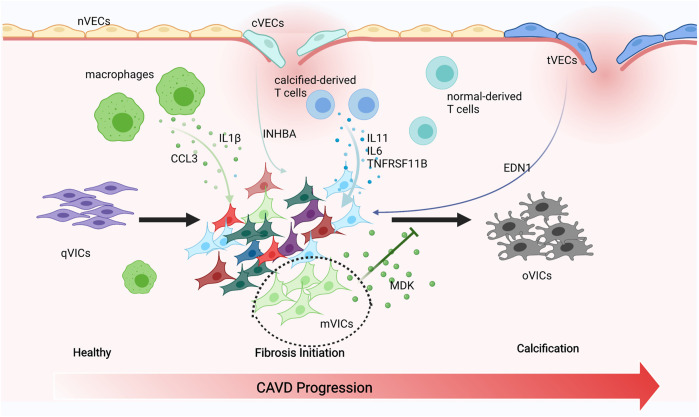
Schematic illustration of intercellular cross talk among valve cells**.** Macrophages secreting IL1β and CCL3 to activate VICs’ subsets. cVECs secreting INHBA and tVECs secreting END1 to affect VICs. Calcified-derived T cells secreting IL11, IL6, and TNFRSF11B to activate VICs. mVICs secreting MDK to inhibit VICs’ calcification.

Endothelial cells tightly cover the surface of the heart valve to avoid the influx of foreign cells and substances into the valve and to maintain valve homeostasis (Takx et al., 2015; Singh et al., 2008; Freeman and Otto 2005). However, VECs’ dysfunction and damage are motivated by hemodynamic changes that initiate the onset and progression of CAVD (Richards et al., 2013; Fernandez Esmerats, Heath, and Jo 2016; Yabusaki et al., 2016). VECs have the unique capacity to undergo EndMT, which plays a crucial role in valve calcification and is important during valvulogenesis (Yu et al., 2014; Kovacic et al., 2019; Balachandran et al., 2011). VECs within adult valves can replenish VICs and reshape the valve leaflets through the EndMT process (Paruchuri et al., 2006). On the other hand, VECs can also contribute to VICs’ calcification through EndMT that is TGFβ-dependent through the inflammation-mediated process (Mahler, Farrar, and Butcher 2013). The diverse VECs’ populations found in our research have been directly linked to EndMT. We observed that nVECs derived from normal valve interact with VICs through the EDN signaling pathway, and tVECs expressed mesenchymal markers interplay with VICs through the ANGPTL signaling pathway, which includes a wide assay of molecules attributed to the TGF family. The comparison between the two indicates that VECs undergo EndMT to achieve VICs’ replenishment through the EDN pathway and to cause VICs’ calcification through the ANGPTL pathway (Figure 7). With the disturbance of endothelium, cVECs derived from calcific valve also raise expression of inflammatory cytokines, which is lined with previous studies (Mahler, Farrar, and Butcher 2013). In summary, effects on EndMT differ from VECs to VECs, which are driven by the particular VECs’ subpopulation through precise approaches.

The pathobiology of CAVD is intricate, encompassing genetic factors, lipid infiltration, and oxidative damage; the complicated immune cell networks are now being accepted to play an essential role in disease continuation (Bartoli-Leonard, Zimmer, and Aikawa 2021). Our research found that there were three types of immune cells in the aortic valve, namely, macrophages, T cells, and DCs. Macrophages have been connected with the progression and severity of valve calcification and atherosclerosis, considered to be crucial drivers of early valve inflammation (Moore and Tabas 2011; Passos et al., 2020). With extracellular matrix remodeling and hemodynamic obstruction, macrophages differentiate into pro-inflammatory (M1) and anti-inflammatory (M2) macrophages (Malyshev and Malyshev 2015). However, only pro-inflammatory macrophages were observed in our study and they produced CCL3, IL1β, and MMP9, all of which attract monocytes to the local region propagate inflammatory responses. On the other hand, pro-inflammatory cytokines such as IL1β were proven to promote osteogenic differentiation of VICs through the NFkB pathway (Chinetti-Gbaguidi et al., 2017; Hjortnaes et al., 2010). Thus, targeted therapies could focus on reducing M1 macrophages, leading to a cell-mediated decrease of calcification. DCs express CCR7 and IDO1 within the calcific valve and present lipid-related antigens to T cells in a way that depends on MHC class II, triggering a pro-inflammatory phenotype, and promoting the development of inflammation and mineralization (Ait-Oufella et al., 2014). Previous reports also demonstrated that DCs were colocalized with oxidized lipid, suggesting a regulatory role associated with lipid infiltration in CAVD (Choi et al., 2009). T cells have long been noted in the aged and calcific valve and even have been considered to be an indicator of aortic stenosis severity (Steiner et al., 2012; Olsson et al., 1994; Otto et al., 1994). More recently, researches have proved that T cells gathered around regions of calcification (Olsson et al., 1994; Otto et al., 1994; Steiner et al., 2012), while their functions in normal human aortic valves tissue have not yet been studied. We observed that IL11, IL6, and TNFRSF11B (OPG) encoding osteoprotegerin, which are inextricably linked to EndMT and VIC calcification, were downregulated in normal-derived T cells (Figure 7) (Rattazzi et al., 2018; Gonzalez Rodriguez et al., 2021). Therefore, we inferred that normal-derived T cells play a vital role in maintaining aortic valve homeostasis and calcific-derived T cells play a crucial role in contributing to CAVD development.

VICs are the foundation for understanding the pathophysiology of CAVD (Rutkovskiy et al., 2017) and actively drive valve calcification by acquiring osteogenic phenotypes (Yip and Simmons 2011). The shift of VICs into osteoclast-like cells is ascribed to pathological stimuli, including endothelial cells injury, chronic inflammation, low-density lipoprotein cholesterol deposition, and reactive oxygen species. VICs were categorized into six subsets, namely, aVICs, cVICs, iVICs, mVICs, liVICs, and sVICs, and every cluster reflected relevant biological functions of disease progression. Identification of VICs’ subsets could provide therapeutic targets to alleviate CAVD. Cell-to-cell interactions rely on the secretion of cytokines that play a crucial role in CAVD, with recent researches underscoring the complexity and interconnectivity of the resident cells (Schlotter et al., 2018). IL6 is implicated as an active driver in valve calcification (Akahori et al., 2018), with IL6 silencing shown to prevent mineralization *in vitro* (El Husseini et al., 2014). IL1β promotes the expression of matrix metalloproteinases (MMPs) (Matilla et al., 2020), both of which exacerbate osteogenic differentiation of VICs and increase the production of inflammatory mediators through the NFkB axis. Further evidence confirmed that IL1β receptor agonist (IL1RA) deficiency could significantly improve the progression of CAVD (Isoda et al., 2010). So far, however, these important cytokines are secreted by which subsets have not been specified. In this study, there were comprehensive descriptions that IL6 is secreted by cVICs and aVICs affecting all VICs’ subsets and IL1β originated from cVICs is critical to the inflammation within the valve (Figure 7).

Impressively, mVICs were the most specific VICs’ subset and highly expressed MDK from bioinformatics analysis. It was the first report that MDK prevented VICs’ calcification, which was demonstrated through the experiment *in vitro*. MDK as a heparin-binding growth factor could interact with different receptors, including syndecans, integrins, protein tyrosine phosphatase ζ, anaplastic lymphoma kinase (ALK), low-density lipoprotein (LDL) receptor-related protein (PRP), and Notch2 receptor (Filippou, Karagiannis, and Constantinidou 2020), and has multifaceted functions, including contribution to diseases development and maintenance of normal tissue homeostasis (Filippou, Karagiannis, and Constantinidou 2020). For example, MDK-Notch signaling, Notch2 as a functional receptor of MDK, regulates the epithelial-mesenchymal transition and chemotherapy resistance in pancreatic cancer (Gungor et al., 2011) and promotes the development of neuroblastoma (Kishida et al., 2013). Therefore, MDK can induce tumorigenesis through Notch signaling. The Notch pathway has an inevitable connection with CAVD because NOTCH1 mutation causes valve calcification (Garg et al., 2005). However, the details of the Notch dysfunction causing CAVD have not been fully clarified. Activation of the Notch pathway seems to restrain VICs’ calcification (Nigam and Srivastava 2009; Acharya et al., 2011). On the other hand, activation of the Notch pathway promotes VICs’ osteogenic differentiation (Zeng et al., 2013), and interactions between VICs and VECs increase NOTCH1 and HEY1 expressions in VICs, accelerating their osteogenic transformation (Kostina et al., 2019). Furthermore, Notch-dependent mechanisms of valve calcification are different in calcific bicuspid and tricuspid aortic valve; VICs, derived from CAVD patients, tend to undergo osteogenic differentiation owing to the activation of the Notch pathway (Kostina et al., 2018). In our study, the expressions of DLL4, NOTCH1, CSL, HES1, and HEY1 were downregulated after treatment of MDK, which indicated that the Notch pathway was suppressed (Supplementary Figure S8). Therefore, we convincingly determined that the MDK-Notch axis plays an important role in preventing human VICs’ osteogenic differentiation (Figure 7).

Taken together, our results provided a comprehensive landscape of cell-to-cell interactions among all cell subsets, which could provide potential reference and guidance for experimental design and may advance the identification of potential therapeutic targets for precision medicine.

## Data Availability

The original contributions presented in the study are included in the article/Supplementary Material; further inquiries can be directed to the corresponding authors.

## Supplementary Material

Supplementary Figure 1 Workflow of single-cell transcriptome analysis and experimental verification.

Supplementary Figure 2 Quality control of scRNA-seq data. **(A)** Density distribution plots reflect the gene numbers, mitochondrial ratio, UMI numbers, and log10 Genes per UMI in each sample. **(B)** The bar plot shows original cell numbers in each sample. **(C)** Cell cycle distribution. **(D)** The plot shows gene numbers versus the number of UMIs colored by the fraction of mitochondrial reads.

Supplementary Figure 3 Differential genes analysis of scRNA-seq and bulk RNA-seq data. **(A–C)** Differential genes were performed as comparing cells from normal and calcified aortic valve tissue within VECs, immune cells, and VICs. The full table of genes is found in the Supplementary Table. **(D–F)** Functional enrichment analysis of significantly upregulated genes in cells from the calcified compared with the normal aortic valve tissue. **(G)** The volcano plot shows the number of upregulated and downregulated genes in bulk RNA-seq data. **(H)** The heatmap shows significantly differential genes. (I) Pathway enrichment of upregulated genes.

Supplementary Figure 4 Characteristics of immune cell. **(A)** The heatmap indicates top 10 maker genes in macrophages, T cells, and DCs. **(B)** Transcription factor activity of these cell types.

Supplementary Figure 5 Cell-to-cell communication patterns. **(A,B)** Heatmaps show the expression level of each pattern genes in each cluster (left), as well as the strength of each pathway gene expression in each pattern (right).

Supplementary Figure 6 Outgoing pathways. **(A,C,E)** Hierarchical plots show inferred intercellular communication networks for ANGPTL, CCL, and IL1 pathways. **(B,D,F)** Heatmaps show the relative importance of each cell subset.

Supplementary Figure 7 Incoming pathways. **(A,C)** Hierarchical plots show inferred intercellular communication networks for IL6 and EDN pathways. **(B,D)** Heatmaps show the relative importance of each cell subsets.

Supplementary Figure 8 RNA expression of genes in the Notch pathway.
