# Supplementary figures and images for: Midkine Prevents Calcification of Aortic Valve Interstitial Cells via Intercellular Crosstalk
Source: Front Cell Dev Biol. 2021 Dec 15;9:794058. doi: 10.3389/fcell.2021.794058 (PMC8714929; doi:10.3389/fcell.2021.794058)

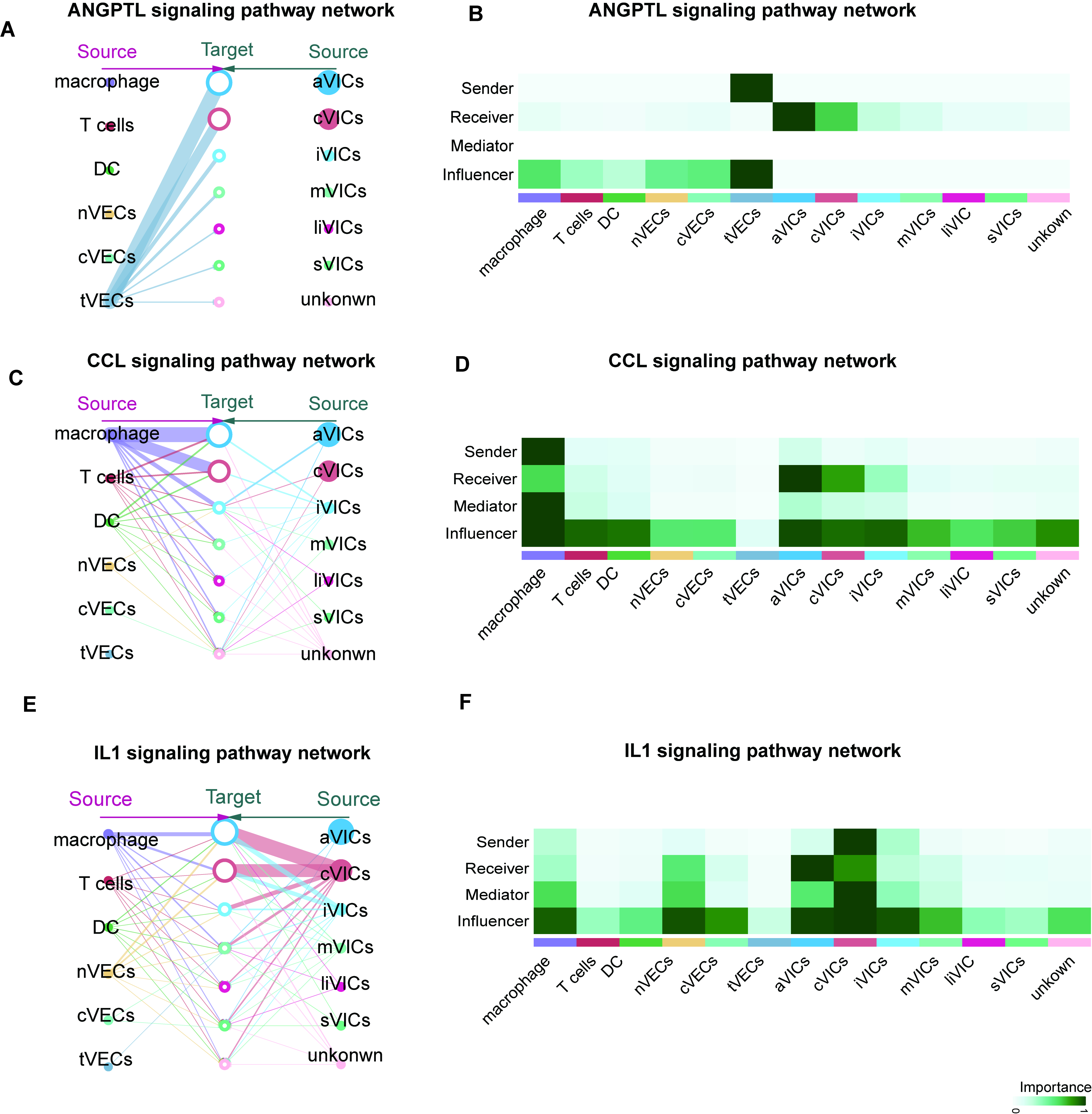

Supplement: Supplementary file 1 [file Image6.TIF]

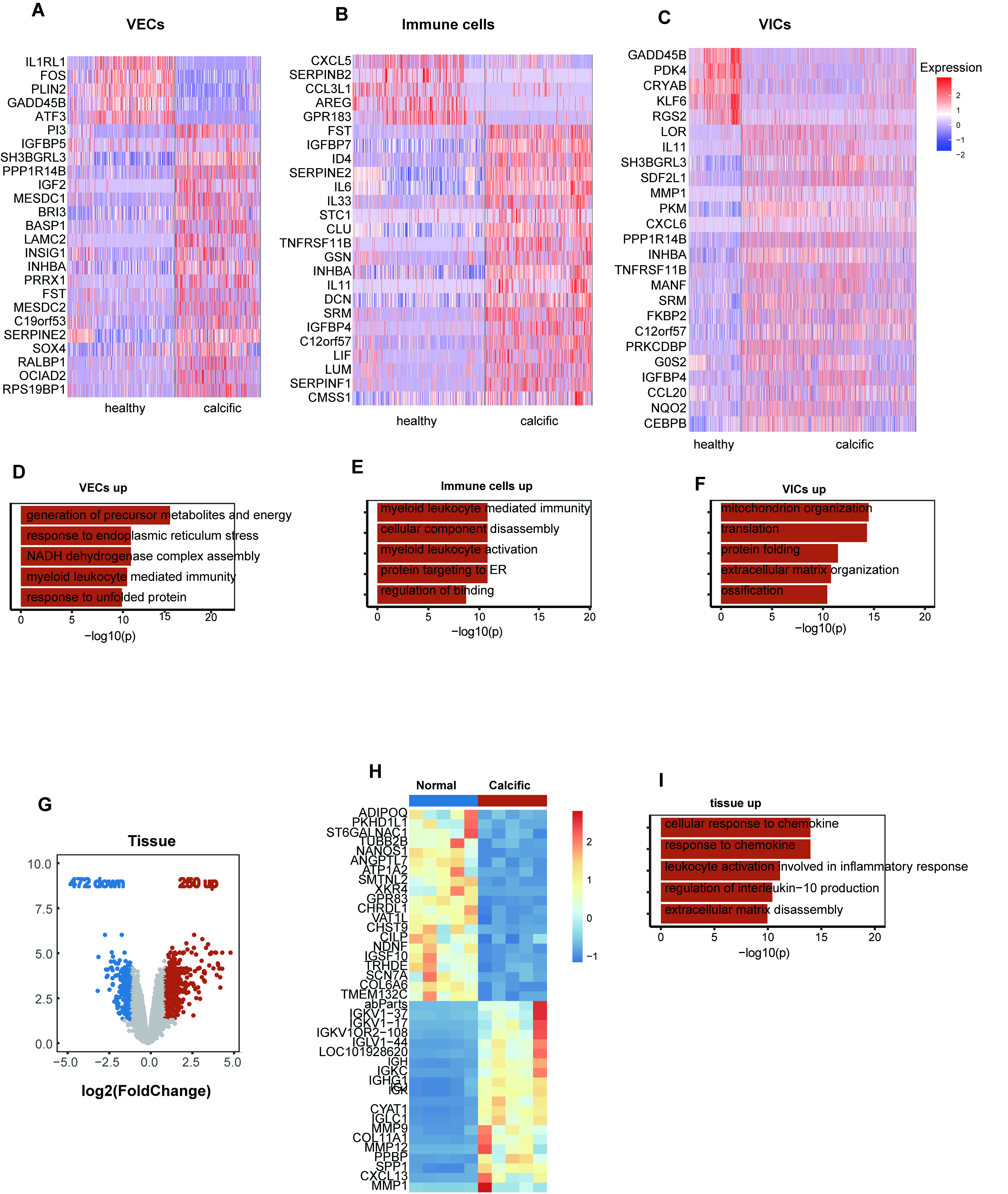

Supplement: Supplementary file 3 [file Image3.TIF]

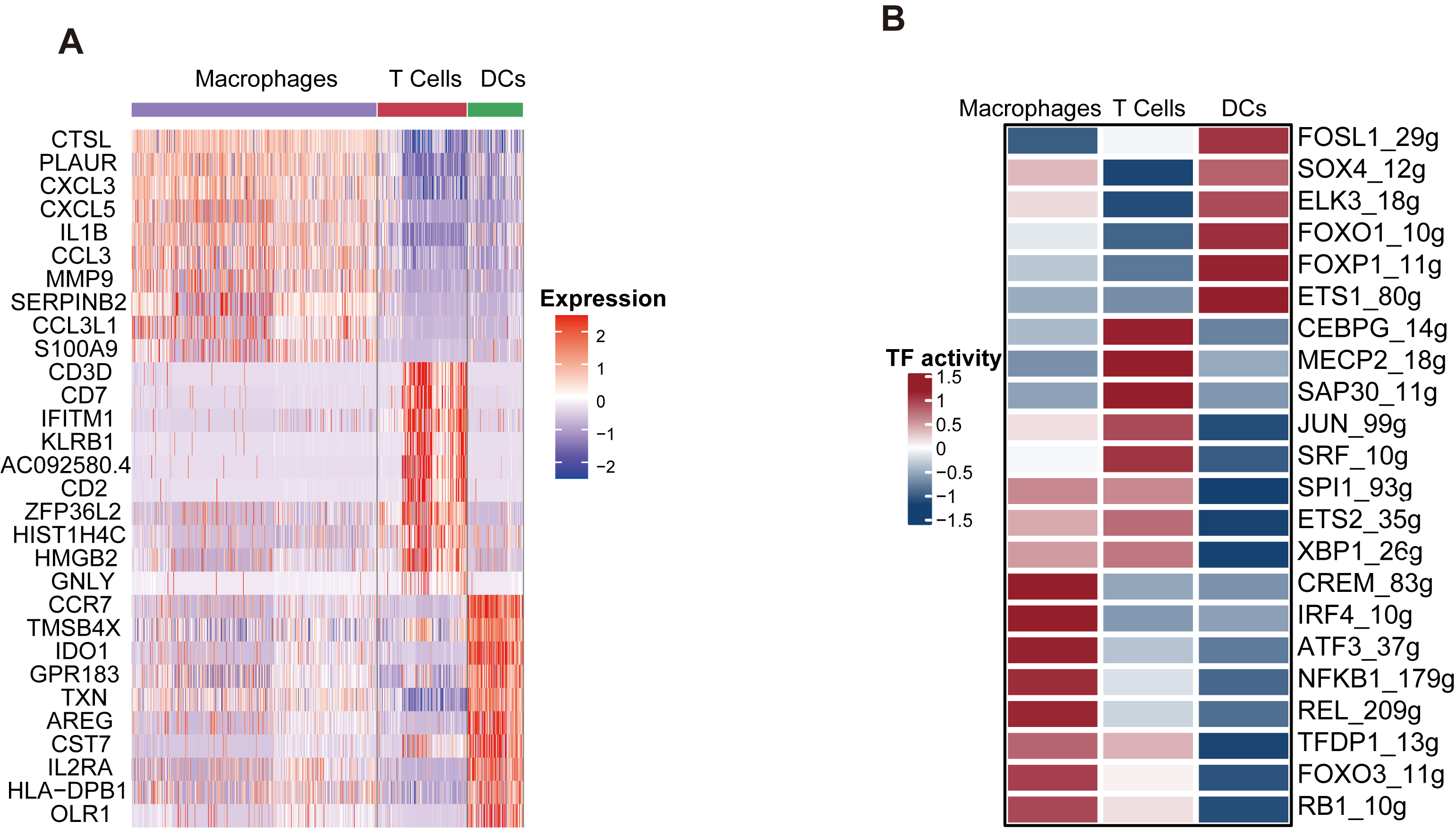

Supplement: Supplementary file 4 [file Image4.TIF]

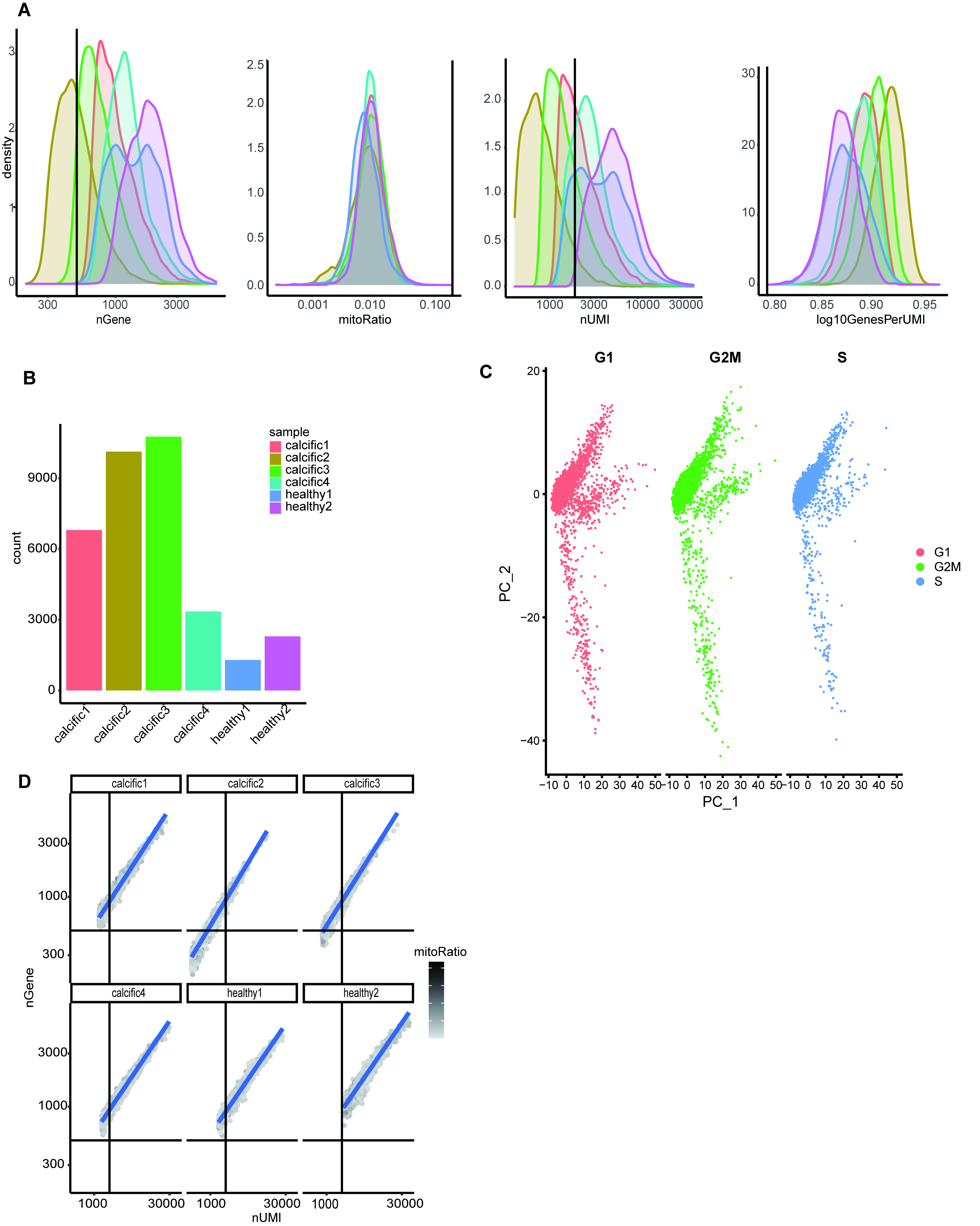

Supplement: Supplementary file 5 [file Image2.TIF]

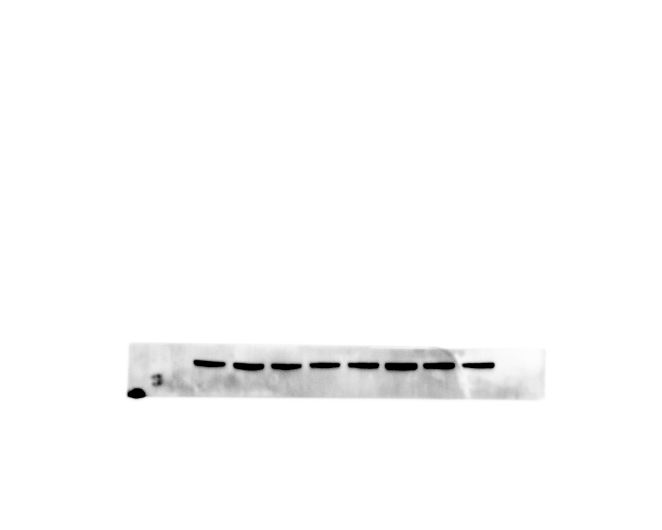


GAPDH

RUNX2


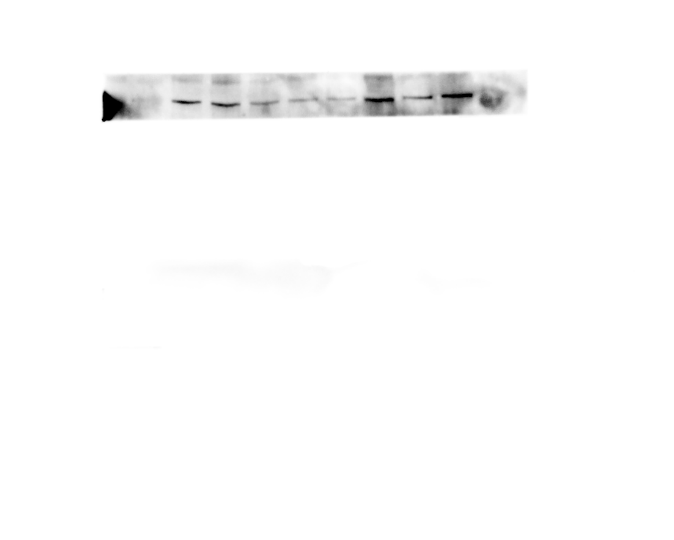


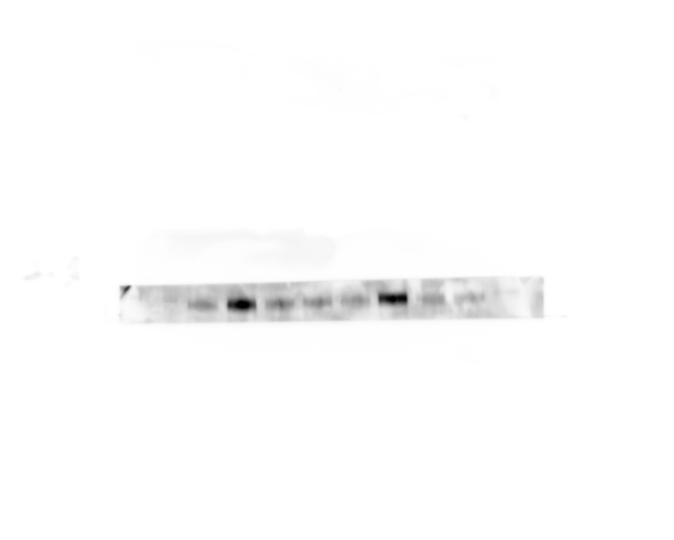


ALP

Supplement: Supplementary file 6 [file DataSheet5.docx]

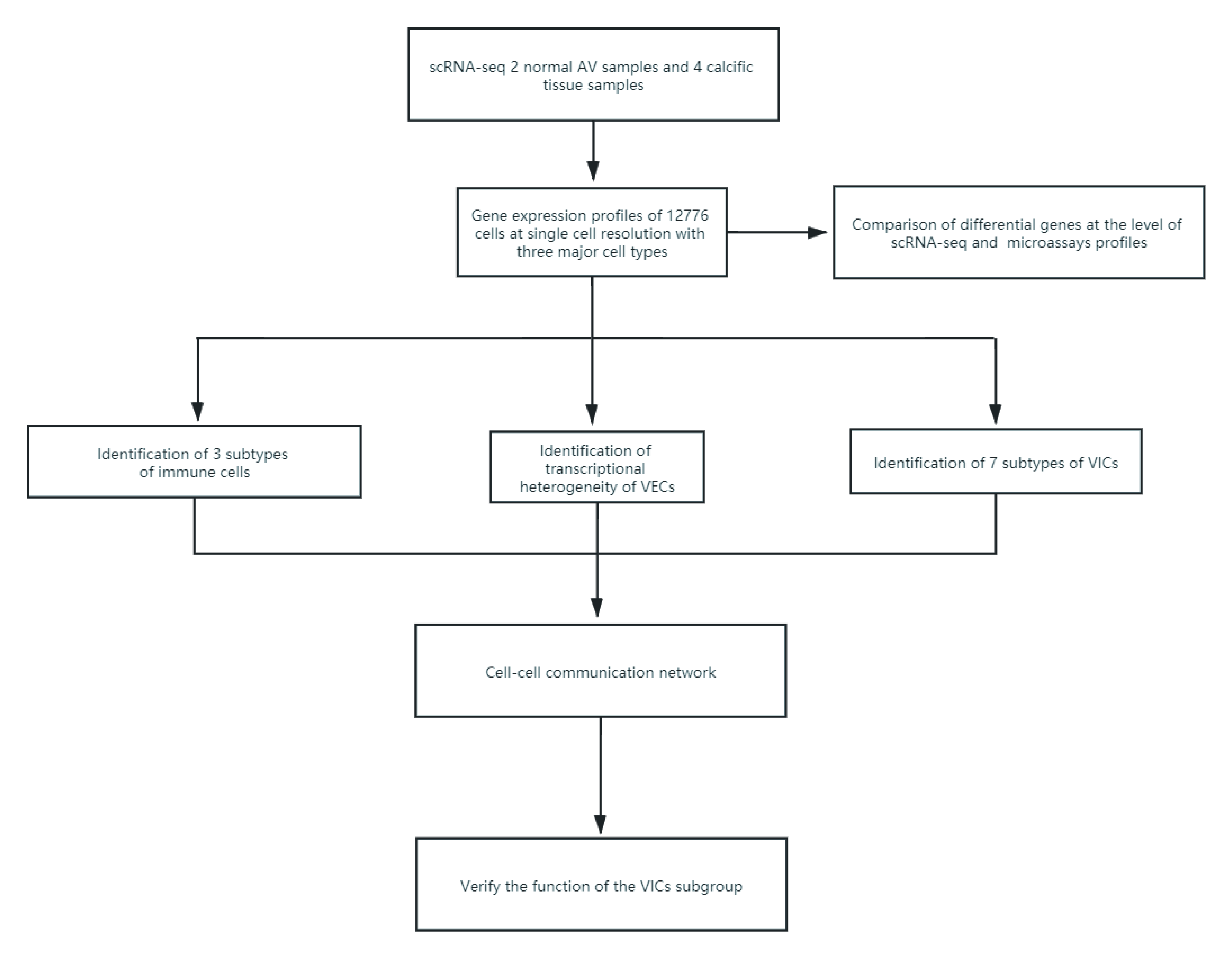

Supplement: Supplementary file 7 [file Image1.TIF]

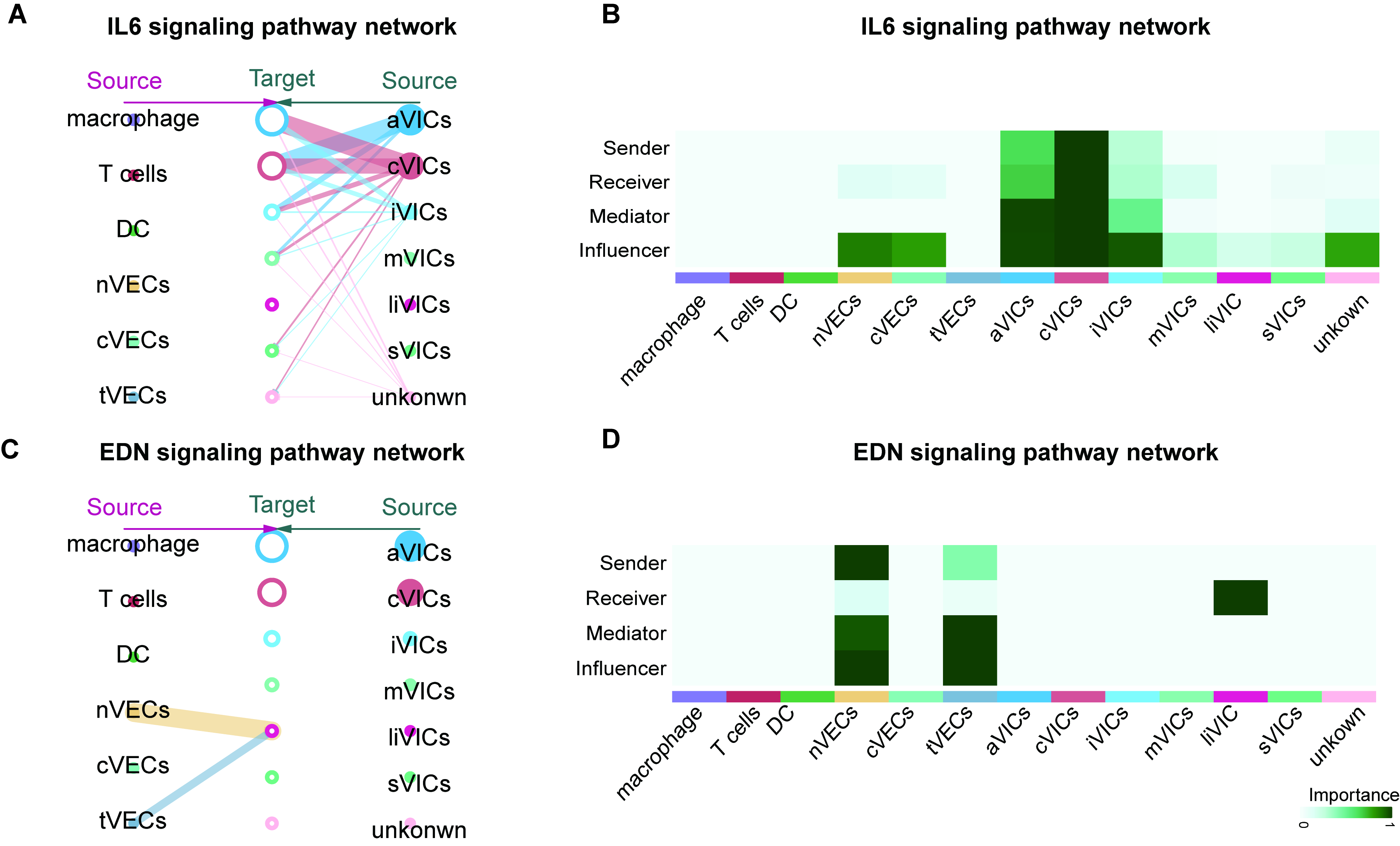

Supplement: Supplementary file 8 [file Image7.TIF]

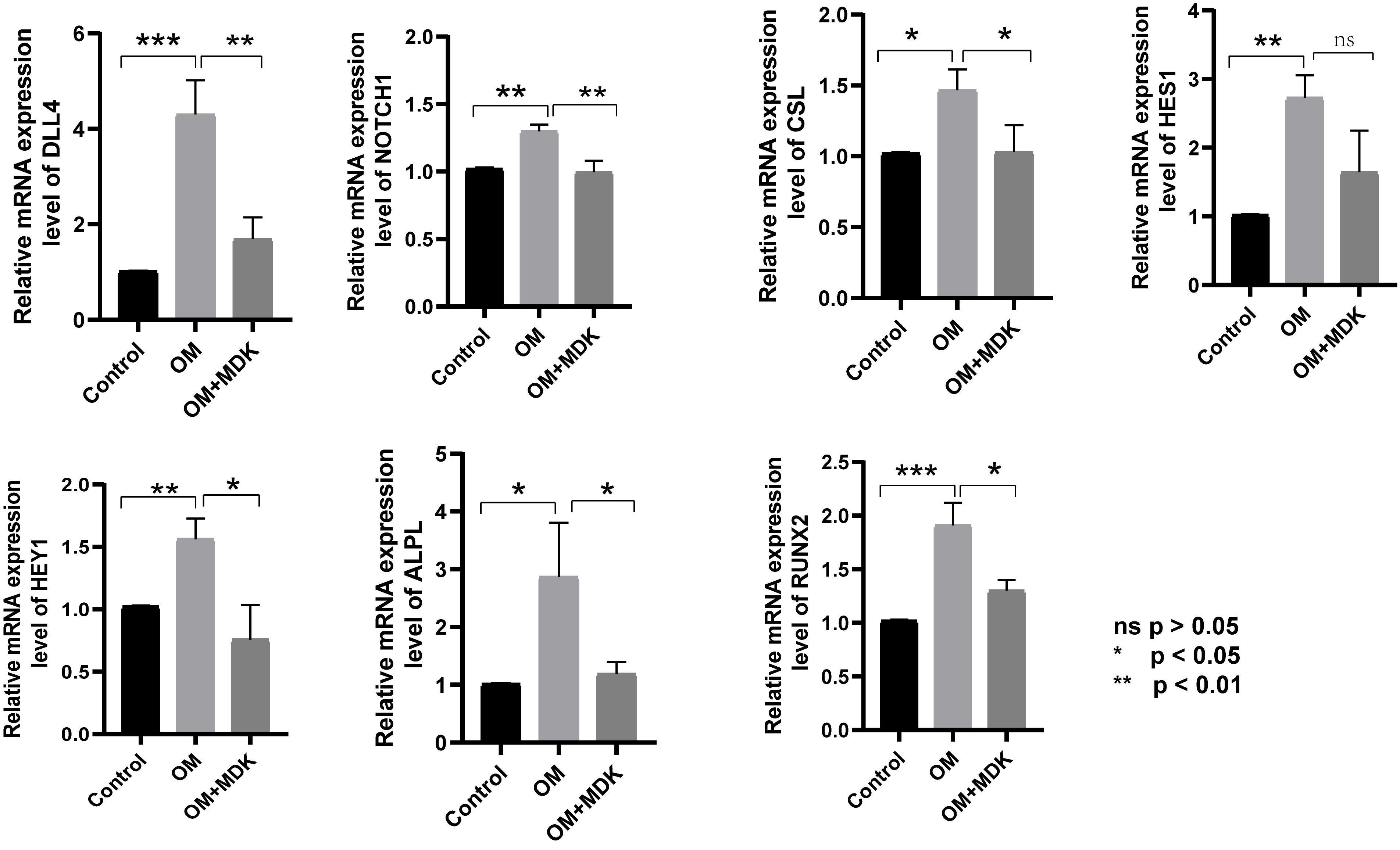

Supplement: Supplementary file 10 [file Image8.TIF]

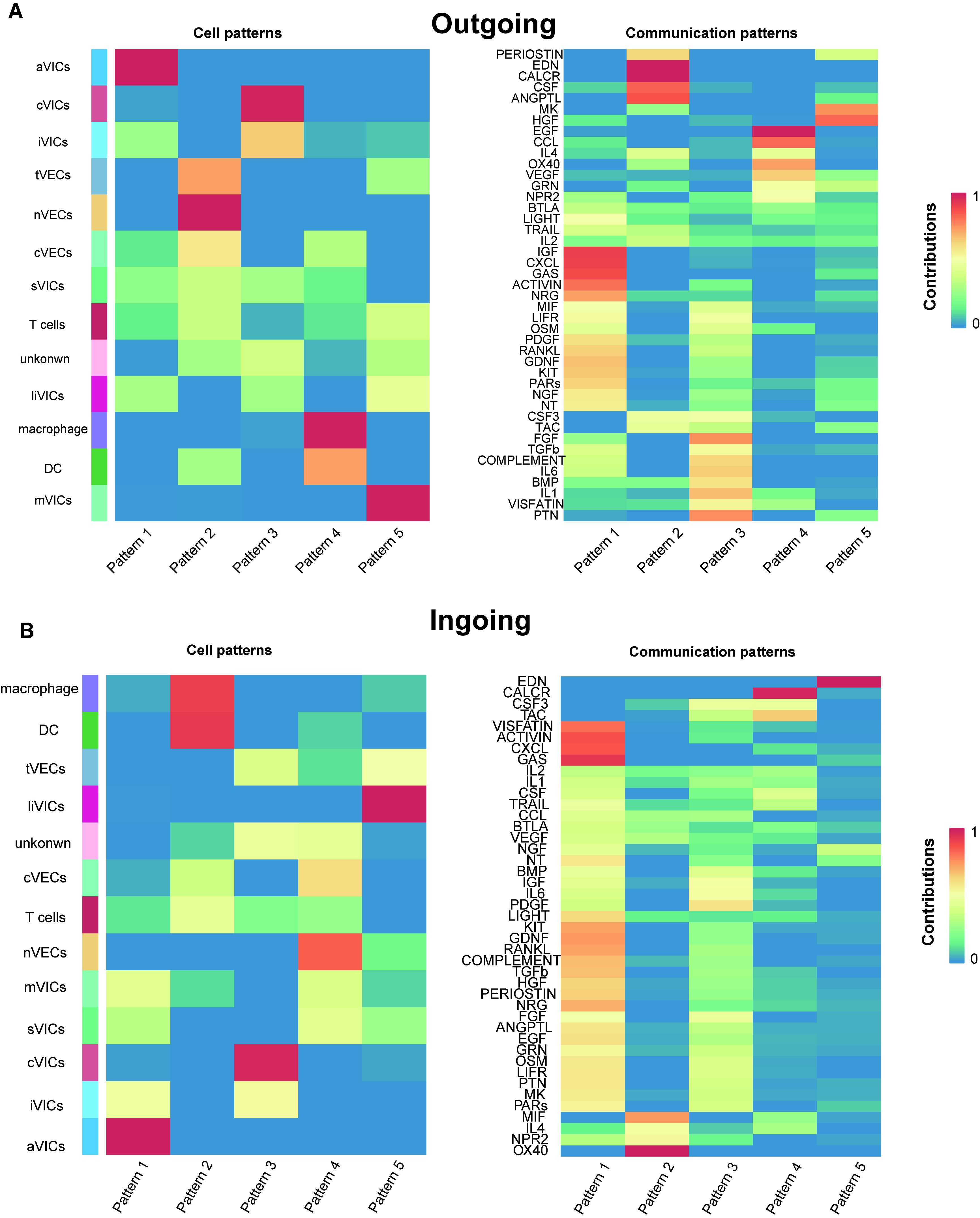

Supplement: Supplementary file 11 [file Image5.TIF]
